# The optimized CO_2_-added ammonia explosion pretreatment for bioethanol production from rice straw

**DOI:** 10.1007/s00449-014-1165-x

**Published:** 2014-03-27

**Authors:** Young-Lok Cha, Jungwoo Yang, Jong-Woong Ahn, Youn-Ho Moon, Young-Mi Yoon, Gyeong-Dan Yu, Gi Hong An, In-Hu Choi

**Affiliations:** Bioenergy Crop Research Center, National Institute of Crop Science, Rural Development Administration, 199 Muan-ro, Cheonggye, Muan, 534-833 South Korea

**Keywords:** Lignocellulosic bioethanol, Pretreatment, Rice straw, Saccharification and fermentation, Response surface methodology

## Abstract

A CO_2_-added ammonia explosion pretreatment was performed for bioethanol production from rice straw. The pretreatment conditions, such as ammonia concentration, CO_2_ loading level, residence time, and temperature were optimized using response surface methodology. The response for optimization was defined as the glucose conversion rate. The optimized pretreatment conditions resulting in maximal glucose yield (93.6 %) were determined as 14.3 % of ammonia concentration, 2.2 MPa of CO_2_ loading level, 165.1 °C of temperature, and 69.8 min of residence time. Scanning electron microscopy analysis showed that pretreatment of rice straw strongly increased the surface area and pore size, thus increasing enzymatic accessibility for enzymatic saccharification. Finally, an ethanol yield of 97 % was achieved via simultaneous saccharification and fermentation. Thus, the present study suggests that CO_2_-added ammonia pretreatment is an appropriate process for bioethanol production from rice straw.

**Electronic supplementary material:**

The online version of this article (doi:10.1007/s00449-014-1165-x) contains supplementary material, which is available to authorized users.

## Introduction

Growing environmental concerns over the depletion of fossil fuels and gradual increase in energy demand have stimulated interest in alternative biofuels, such as bioethanol, over the last few decades [1]. Bioethanol is mainly of interest as a petrol additive or substitute because ethanol-blended fuel undergoes cleaner and more complete combustion that reduces greenhouse gas and toxic emissions [2]. As a consequence of the surge in demand for bioethanol, lignocellulosic biomass has recently attracted attention for bioethanol production and one of examples is rice straw which is the most abundant lignocellulosic biomass worldwide [3]. However, one of the primary factors for bioethanol production is ensuring a stable supply of the biomass [4].

Lignocellulosic biomass is generally defined as the materials that compose the plant cell wall, primarily consisting of cellulose, hemicellulose, and lignin. These polymeric complexes are resistant to degradation [5]. Thus, the fundamental principle of biomass pretreatment includes swelling, phase change in cellulose crystallinity, and removal of lignin under ambient/high temperature and pressure with buffering [6]. Pretreatment is considered as a central process for bioethanol production because the pretreatment step is known to be the most expensive and profoundly affects all downstream steps, such as enzyme hydrolysis, fermentation, waste residue handling, and ethanol recovery [7]. Pretreatment remains a bottleneck in the process of lignocellulosic bioethanol production, even though various pretreatment methods have been intensively introduced so far [8, 9]. Recently, a combined pretreatment exhibited a synergistic effect for cellulose recovery and enzymatic sugar conversion [10–12].

In this study, a combined pretreatment, CO_2_-added ammonia explosion, was performed for bioethanol production from rice straw based on ammonia fiber explosion (AFEX) [13], soaking in aqueous ammonia (SAA) [14, 15], and carbon dioxide explosion (CDE) pretreatments [16]. Each method was independently proven to increase the sugar conversion yield and thereby to increase ethanol yield with varying temperature (25–200 °C), pressure (1,000–4,000 psi), and residence time (5 min–72 h) [17]. Reagent ammonia is known not only to induce swelling of lignocellulosic materials but also to remove lignin [13], whereas carbon dioxide is known to penetrate the biomass under high pressure, resulting in pore size increase in the lignocellulosic complex [16]. Residual ammonia from AFEX or SAA pretreatment is reported to enrich the pretreated lignocellulosic biomass [13], and CO_2_ can be collected during fermentation and recycled for various uses [18]. Thus, CO_2_-added ammonia explosion was optimized using response surface methodology (RSM) with regard to CO_2_ recycling for further investigation and synergic pretreatment effects. Finally, mass balance analysis was performed to evaluate the efficacy of the combined pretreatment following simultaneous saccharification and fermentation (SSF).

## Materials and methods

### Rice straw

Rice straw (*Oryza sativa L.*) was obtained from Muan, Jeonnam, Korea in 2010. The air-dried rice straw was chopped to a length of 5 cm using a tub grinder (Tomotech Ltd.; Korea). The chopped rice straw was then ground using a 20-hp hammer mill (Sunbrand Industrial Inc.; Korea) with 1.0-mm screens, dried in an oven at 60 °C for 24 h, and then stored in desiccators. The chemical analysis indicated that the rice straw mainly consisted of 31.8 wt% cellulose, 17.5 wt% hemicellulose, 18.2 wt% lignin, and 6.9 wt% ash.

### Pretreatment

**Fig. 1:**
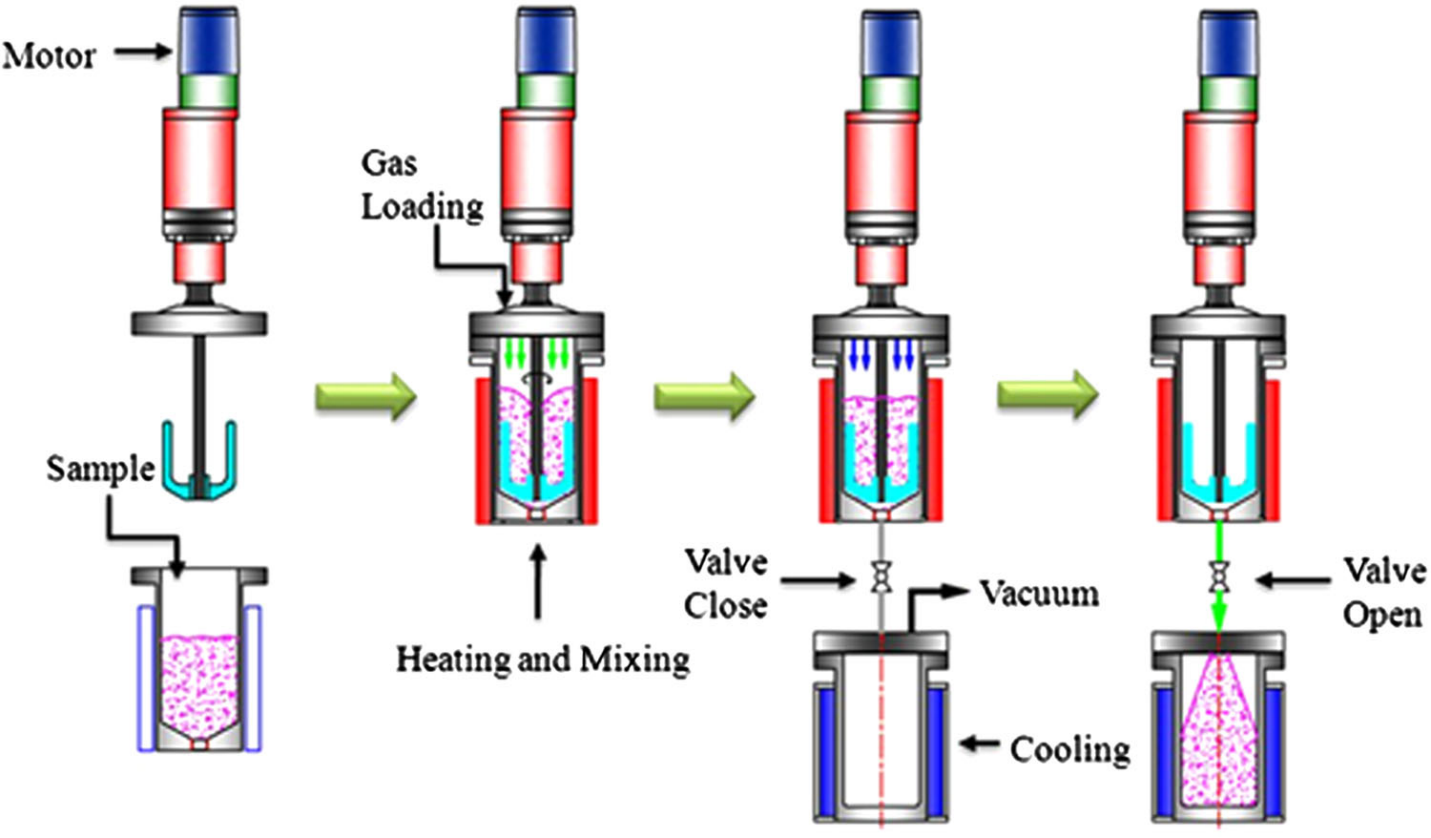
Schematic description of the pretreatment process

The pretreatment was performed in a 800-ml pressure vessel equipped with a temperature and pressure sensor (Fig. 1). After the mixture of rice straw and aqueous ammonia (1:14, 300-ml working volume) was loaded into the vessel, pressurized CO_2_ gas was loaded up to 0–3.0 MPa. The vessel was then heated to 130–190 °C for 10–90 min. 6 MPa of nitrogen gas was additionally loaded into the vessel for explosion before the pretreated rice straw was collected into a separator via pressure and temperature differences. The solid hydrolysate was obtained using a Buchner funnel with a 10-μm nylon filter and neutralized with tap water.

### Response surface methodology (RSM)

To optimize important variables affecting the combined pretreatment RSM was used. A four-factor factorial central composite design (CCD) was constructed under the following conditions: temperature of 130–190 °C, residence time of 10–90 min, ammonia concentration of 0–20 %, and CO_2_ pressure of 0–3.0 MPa (Table 1). Thirty combinations of these parameters were tested, and the significance of each variable and interactions between variables was evaluated by analysis of variance (ANOVA). The optimal conditions were determined on the basis of the degree of glucose recovery following enzymatic hydrolysis. Finally, the response surface regression of the acquired data was analyzed using Design Expert software version 8.1.

**Table 1:** Central composite design matrix and results from the measured responses

Run	Factors				Total glucose conversion rate (%)
	Temperature (^o^C)	Time (min)	Ammonia concentration (%)	CO_2_ loading (MPa)	
Avicel	–	–	–	–	97.32
1	145	30	5	0.75	77.99
2	175	30	5	0.75	90.19
3	145	70	5	0.75	84.46
4	175	70	5	0.75	89.16
5	145	30	15	0.75	90.48
6	175	30	15	0.75	99.04
7	145	70	15	0.75	93.39
8	175	70	15	0.75	93.12
9	145	30	5	2.25	69.36
10	175	30	5	2.25	69.05
11	145	70	5	2.25	81.96
12	175	70	5	2.25	92.81
13	145	30	15	2.25	79.87
14	175	30	15	2.25	97.18
15	145	70	15	2.25	88.04
16	175	70	15	2.25	98.62
17	130	50	10	1.50	84.80
18	190	50	10	1.50	97.48
19	160	10	10	1.50	87.43
20	160	90	10	1.50	93.04
21	160	50	0	1.50	46.75
22	160	50	20	1.50	97.82
23	160	50	10	0	96.00
24	160	50	10	3.00	95.95
25	160	50	10	1.50	97.12
26	160	50	10	1.50	88.76
27	160	50	10	1.50	96.37
28	160	50	10	1.50	85.44
29	160	50	10	1.50	97.60
30	160	50	10	1.50	87.64

### Simultaneous saccharification and fermentation (SSF)

The industrial yeast strain *Saccharomyces cerevisiae* CHY 1011 was kindly provided by Changhae R&D [19]. The cells were maintained at 30 °C in YPD (1 % yeast extract, 2 % peptone, and 2 % glucose and 1.5 % agar for solid plates). SSF was conducted similar to the procedure described in the National Renewable Energy Laboratory (NREL) LAP-009 [20] and LAP-008 [21]. Briefly, the solid hydrolysate containing 3 % glucan (g/v) was transferred into a 250-ml flask containing 0.05 M citrate buffer at pH 4.8. Then, 20 FPU/g cellulase (Novozymes; Cellic Ctec II), 2 % peptone, 1 % yeast extract, and distilled water were additionally loaded to give a working volume of 100-ml. Finally, preconditioned yeast cells were harvested from the 100-ml culture and inoculated into the flask when the cell density was approximately optical density (OD_600_ = 4.0). SSF was then performed at 33 °C for 72 h with an agitation speed of 150 rpm. Samples were taken periodically to determine ethanol production and sugar consumption.

Enzymatic hydrolysis was performed at 50 °C for 24 h with 20 FPU/g cellulase (Novozymes; Cellic Ctec II).

### Analytical methods

A compositional analysis of pretreated and unpretreated rice straw was conducted according to NREL LAP-002 [22] and LAP-003 [23]. Sugar concentrations were determined by high-performance liquid chromatography (HPLC; Waters Corporation, USA). Briefly, samples filtered using a 0.2-μm membrane were loaded in an Aminex HPX-87H column (Bio-Rad; Hercules, CA, USA) set to 65 °C and eluted with 0.5 mM H_2_SO_4_ at a constant flow rate of 0.6 ml/min. Peaks were detected using a refractive index detector and quantified according to a calibration curve. The ethanol concentration during SSF was determined by gas chromatography (GC) (Agilent 6980N; Wilmington, DE, USA) equipped a HP-INNOWaX 19091N-133 column at a flow rate of 15 ml/min for the carrier helium gas. Ash content was determined based on the oven-dry method [24], and moisture content was analyzed using a moisture analyzer (HR83 halogen moisture analyzer; Mettler-Toledo; Switzerland).

### Scanning electron microscopy (SEM)

Field emission SEM (TM-100; Hitachi; Tokyo, Japan) was used to observe morphological changes of rice straw. Samples were mounted on aluminum stubs and observed under vacuum conditions at an acceleration voltage of 15 kV without coating.

## Results and discussion

### Effect of CO_2_ addition on the pretreatment of rice straw

**Fig. 2:**
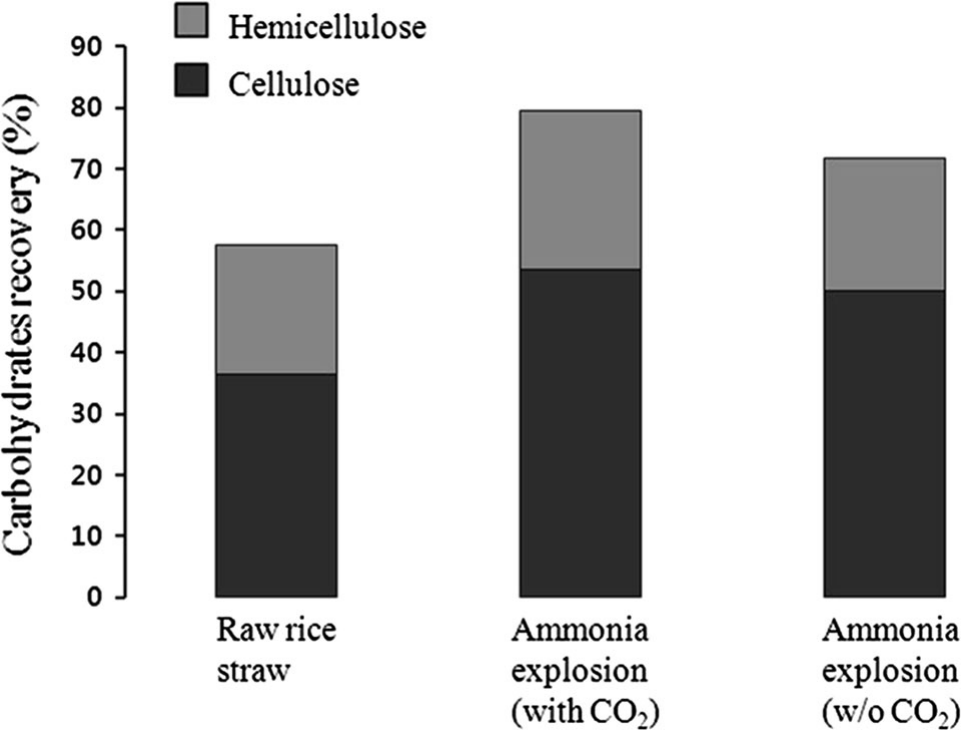
Cellulose and hemicellulose recovery by ammonia explosion with or without pressurized CO_2_ loading

It was initially hypothesized that addition of high-pressurized CO_2_ loading to the ammonia explosion pretreatment could allow effective penetration of the biomass, resulting in a significant increase in enzymatic hydrolysis [16, 25, 26]. Thus, CO_2_ was considered to be useful for pretreatment because the CO_2_ consumed could be recycled. The pretreatment was conducted under conditions of an ammonia concentration of 15 % at 160 °C for 60 min with or without CO_2_ loading. The total yield of recovered cellulose and hemicellulose that could be converted into fermentable sugar was 79.4 wt% in the CO_2_-added pretreatment and 71 wt% without CO_2_ loading (Fig. 2). Solids residues were approximately 58 % in both pretreatment. Although no marked difference was observed in the hydrolysate pretreated with and without CO_2_ loading, it is expected that the difference will be amplified during the pretreatment on a larger scale. Thus, ammonia explosion pretreatment was conducted with CO_2_ loading. However, it would be argued that for economically viable process, it is required to consider additional energy cost caused by high pressure CO_2_ prior to the concept of CO_2_ addition.

### Optimization of pretreatment conditions by RSM for maximal ethanol yield

A four-variable central composite RSM design was used to model optimal pretreatment conditions for rice straw. The independent variables and their ranges were as follows: temperature of 130–190 °C, residence time of 10–90 min, ammonia concentration of 0–20 %, and CO_2_ loading level of 0–3.0 MPa. The total glucose conversion rate (%) from pretreated rice straw was chosen as the outcome for analysis. The 30 runs and responses are summarized in Table 1. Following the pretreatment, compositional changes of the solid hydrolysate were observed as follows: 31.8 wt% cellulose to 41.5–57.6 wt%, 17.5 % hemicellulose to 17.2–23.7, 18.2 wt% of lignin to 7.3–15.5, and 6.9 wt% ash to 10.4–14.0 wt% (Supplementary Table 1). Overall, cellulose content was significantly increased, whereas lignin content was slightly decreased. For saccharification, 20 FPU/g cellulase (Novozymes; Cellic Ctec II) was added to the hydrolysate, which contained 3 % glucan on a dry weight basis. The conversion rate from the hydrolysate varied from 69.1 to 99.0 %, whereas that from Avicel as a control was 97.3 % (Supplementary Table 2). Consequently, the glucose yield based on the reaction conditions was modeled as follows:

$$\begin{gathered} Y{ = 92}. 1 5 5 { } + { 3}. 70 8X_{ 1} + { 2}. 4 8 4X_{ 2} + { 7}. 7 8 8X_{ 3} {-}{ 1}. 7 10X_{ 4} {-} \, 0. 7 4 4X_{ 1} X_{ 2} {-} \, 0. 5 4 6X_{ 1} X_{ 3} \hfill \\ + \, 0. 8 2 8X_{ 1} X_{ 4} {-}{ 2}. 20X_{ 2} X_{ 3} + { 2}. 7 2 1X_{ 2} X_{ 4} + { 1}.0 1 9X_{ 3} X_{ 4} {-} \, 0. 2 9 4X_{1}^{2} {-} \, 0. 5 2X_{2}^{2} {-}{ 5}.00 7X_{ 3}^{ 2} \hfill \\ {-} \, 0. 9 1 5X_{4}^{2} \hfill \\ \end{gathered}$$

where *Y* is glucose yield (%), *X*_1_ is temperature (^o^C), *X*_2_ is residence time (min), *X*_3_ is ammonia concentration (%), and *X*_4_ is CO_2_ loading level (MPa).

**Fig. 3:**
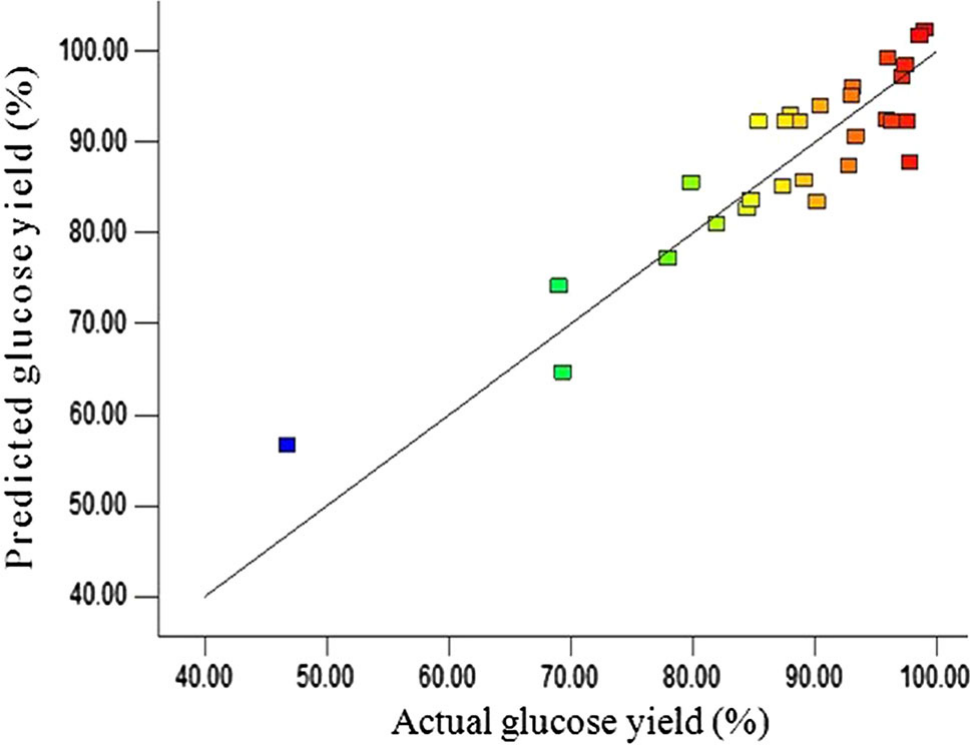
Relationship between predicted glucose yield and actual glucose yield

**Fig. 4:**
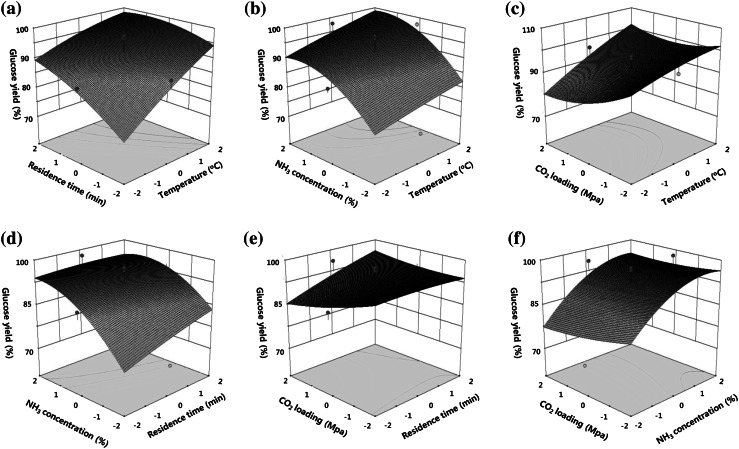
Response surface plots of glucose yield obtained from 30 independent tests. **a** Residence time (min) and temperature (°C); **b** NH_3_ concentration (%) and temperature (°C); **c** CO_2_ loading (MPa) and temperature (°C); **d** NH_3_ concentration (%) and residence time (min); **e** CO_2_ loading (MPa) and residence time (min); **f** CO_2_ loading (MPa) and NH_3_ concentration (%)

To examine the validity of the model ANOVA was performed, and the results are presented in Table 2. An obtained *F* value of 5.12 with a lower *P* value of 0.0017 implied that the model was highly significant. At the same time, the *R* ^2^ value between actual and predicted glucose yield was 0.8268, suggesting that experimental data were correlated with the predicted data to some degree, as shown in Fig. 3. Prob > *F* value less than 0.05 indicates that model terms are significant. The model terms *X*_1_ (temperature), *X*_3_ (ammonia concentration), and *X*_3_^2^ were found to have a significant effect on glucose yield. In spite of the lack of significance of the interactions among variables (*P* > 0.05), these factors were not excluded because of supporting the hierarchy of the model. The interactions of each variable are plotted in Fig. 4. Increased pretreatment temperature with a longer residence time gave an increased percentage of glucose recovery (Fig. 4a). Higher ammonia concentration increased glucose recovery irrespective of CO_2_ loading level or residence time (Fig. 4d, f). When temperature and ammonia concentration increased, glucose yield also increased (Fig. 4b). There was no obvious effect of CO_2_ loading and residence time (Fig. 4e). However, ammonia concentration on pretreatment effect was highly correlated with CO_2_ loading. Overall, glucose yield was significantly increased when ammonia and CO_2_ concentrations were increased (Fig. 4f). However, the CO_2_ loading effect was increased when the temperature was decreased (4 °C). Finally, the predicted optimal pretreatment conditions for maximal glucose yield were determined as follows: temperature, 165.1 °C; residence time, 69.8 min; ammonia concentration, 14.3 %; and CO_2_ loading level, 2.2 MPa. As a result, 27.1 g of glucan was recovered from 51.2 g of pretreated solid hydrolysate from 100 g of raw rice straw containing 25.4 g of glucan.

**Table 2:** ANOVA of the adjusted model from 30 independent pretreatments and enzymatic hydrolysis

Source	Sum of squares	*Df*	Mean square	*F* value	*P* value (Prob > *F*)
Model	3,004.3110	14	214.5936	5.1152	0.0017
*X*_1_ (temp.)	329.8934	1	329.8934	7.8636	0.0133
*X*_2_ (time)	148.1060	1	148.1060	3.5304	0.0798
*X*_3_ (NH_3_)	1,455.4835	1	1455.4838	34.6942	<0.0001
*X*_4_ (CO_2_)	70.1784	1	70.1784	1.6728	0.2154
*X*_1_·*X*_2_	8.8506	1	8.8506	0.2110	0.6526
*X*_1_·*X*_3_	4.7742	1	4.7742	0.1138	0.7405
*X*_1_·*X*_4_	10.9561	1	10.9561	0.2612	0.6168
*X*_2_·*X*_3_	77.4400	1	77.4400	1.8459	0.1943
*X*_2_·*X*_4_	118.4832	1	118.4832	2.8243	0.1135
*X*_3_·*X*_4_	16.6056	1	16.6056	0.3958	0.5387
$X_{1}^{2}$	2.3634	1	2.3634	0.0563	0.8156
$X_{2}^{2}$	7.4107	1	7.4107	0.1766	0.6802
$X_{3}^{2}$	687.7157	1	687.7157	16.3930	0.001
$X_{4}^{2}$	22.9743	1	22.9743	0.5476	0.4707
Residual	629.2762	15	41.9517		
Lack of fit	480.2082	10	48.0208	1.6107	0.3122
Pure error	149.0680	5	29.8136		
Cor total	3,633.5872	29			

*Df* degrees of freedom

The optimal pretreatment was confirmed by performing enzymatic hydrolysis. Enzymatic hydrolysis with pretreated miscanthus containing 3 % glucan was conducted at 50 °C with 20 FPU/g cellulase for 72 h. The converted glucose concentration obtained from optimally pretreated hydrolysate was 31.2 ± 0.2 g/l on average, representing a conversion ratio of 93.6 %. Previously, Kim et al. [27] reported that 87.2 % of glucose yield was achieved with dilute sulfuric acid and aqueous ammonia pretreatment under the conditions of 42.75 °C, 20 % ammonia, and 48 h. Another combined ammonia pretreatment with ionic liquid was carried out with 20 % ammonia at 100 °C for 6 h, and its glucose yield by saccharification was 97 % [12]. In addition, various combined pretreatment based on ammonia pretreatment resulted in enhanced enzymatic hydrolysis up to 90.7 % at optimal conditions, such as temperature, residence time, pressure, enzyme dosage, and biomass size etc. [11, 15, 26, 28]. Thus, our combined pretreatment method to yield 93.6 % of theoretical maximal fermentable glucose might be reasonable for fermentation.

### SSF using the optimally pretreated hydrolysate

**Fig. 5:**
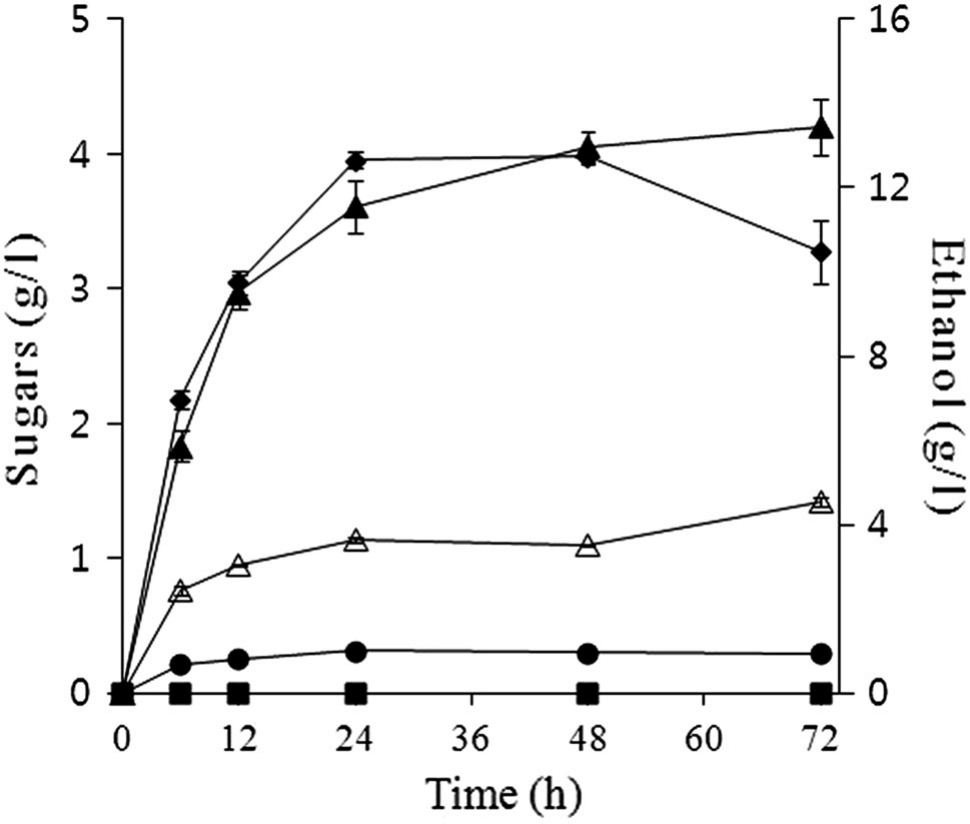
Kinetics of simultaneous saccharification and fermentation (SSF). Ethanol (*filled triangle*), glucose (*filled square*), xylose (*filled diamond*), and arabinose (*filled circle*) from pretreated rice straw; ethanol (*empty triangle*) from unpretreated rice straw

Simultaneous saccharification and fermentation was performed in a 250-ml flask with solid hydrolysate containing 3 % glucan under conditions of 33 °C and 150 rpm for 72 h. The fermentation kinetics is shown in Fig. 5. Ethanol from untreated rice straw reached the saturation point (3.64 ± 0.07 g/l) in 24 h, whereas the amount of ethanol from treated rice straw increased to 13.4 ± 0.66 g/l in 72 h, and the ethanol yield was 97 %. The glucose concentration was constant at 0 % because the fermentation rate would be faster than the saccharification rate at 33 °C. Five-carbon sugars, such as xylose and arabinose, were not notably produced from the hydrolysate because cellulase was used as the enzyme. The limitation of the SSF in this study may be that the yeast strain was not thermo-tolerant and cannot ferment five-carbon sugars. Thus, for efficient ethanol production, further investigations are necessary (e.g., with thermo-tolerant strains or high solid loadings of pretreated hydrolysate).

### SEM analysis

**Fig. 6:**
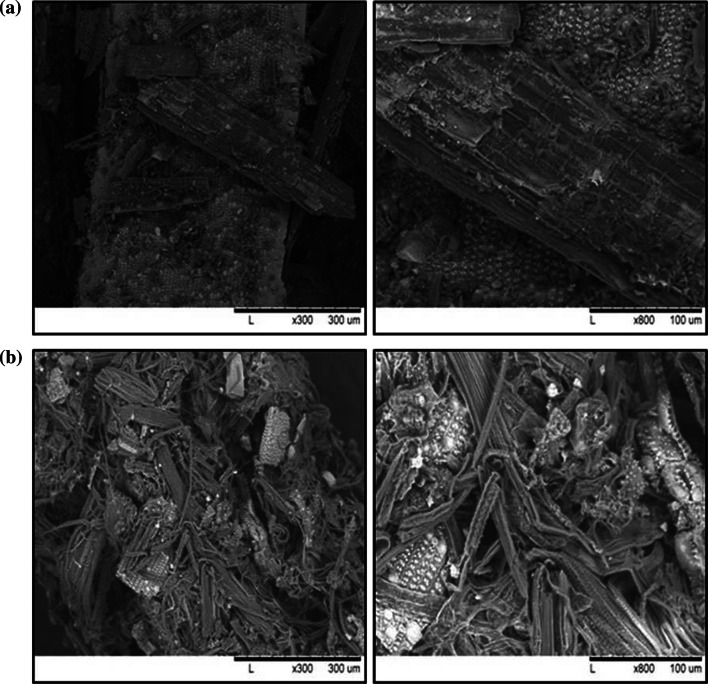
SEM analysis. Photos of untreated (**a**) and treated (**b**) rice straw (×300 and ×800)

Scanning electron microscopy analysis was conducted to determine the morphological changes of pretreated rice straw. As shown in Fig. 6a, untreated rice straw showed a compacted surface structure in the cell wall because of tight bonding between particles. By contrast, cellulose fibers were exposed and scattered throughout the pretreated rice straw, and a few bundles existed in a cracked form (Fig. 6b). This destruction by pretreatment seems to increase enzyme accessibility and enzymatic hydrolysis [29].

### Mass balance analysis

**Fig. 7:**
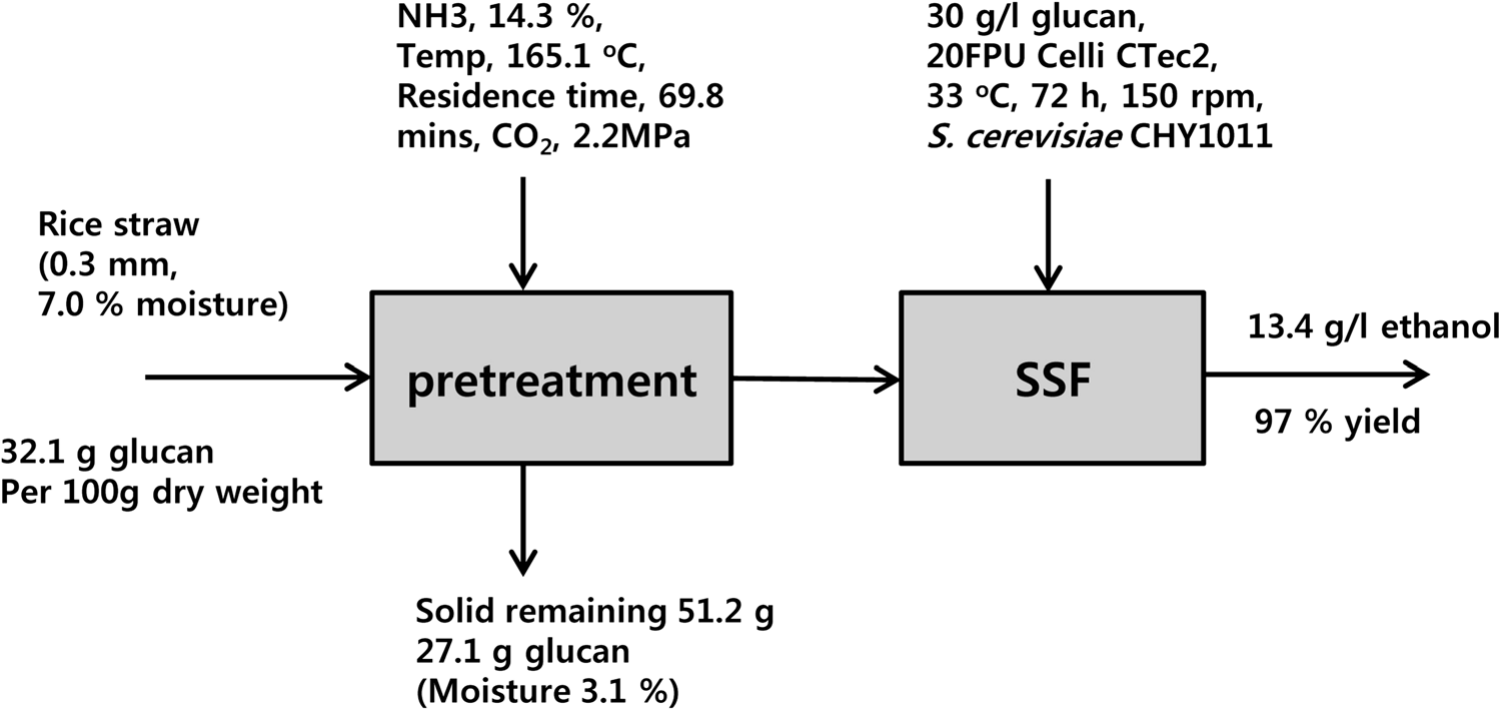
Overall mass balance

The overall mass balance for the pretreatment and SSF is shown in Fig. 7. Initially, the effect of CO_2_ addition to ammonia pretreatment was shown to increase carbohydrate recovery by up to 8 %. Next, the pretreatment conditions were optimized by RSM to obtain the following: 14.3 % for ammonia concentration, 2.2 MPa for CO_2_ loading, 165.1 °C for temperature, and 69.8 min for residence time. The solid content was 51.2 % after pretreatment, and the glucan content was 27.1 g. The glucose yield by enzymatic hydrolysis was up to 93.6 % from the pretreated solids containing 3 % glucan (g/g). In SSF, an ethanol yield of 97 % was achieved; 13.4 g/l from the initial glucan content of 3 %.

## Conclusions

Rice straw was attractive biomass for bioethanol production due to its abundance, but pretreatment process is essential to acquire fermentable sugars from rice straw. In this study, novel pretreatment equipment was designed, fabricated, and applied to CO_2_ -added ammonia explosion pretreatment. The combined pretreatment was optimized and modeled by RSM. The conditions were 14.3 % for ammonia concentration, 2.2 MPa for CO_2_ loading, 165.1 °C for temperature, and 69.8 min for residence time. Our model was verified by enzymatic saccharification, resulting in the glucose yield 93.6 % from rice straw. Finally, bioethanol via SSF could be obtained up to 97 % of theoretical yield.

## Electronic supplementary material

Below is the link to the electronic supplementary material.

Supplementary material 1 (DOCX 19 kb)
