# Supplementary material for: The optimized CO2-added ammonia explosion pretreatment for bioethanol production from rice straw
Source: Bioprocess Biosyst Eng. 2014 Mar 27;37(9):1907–15. doi: 10.1007/s00449-014-1165-x (PMC4141972; doi:10.1007/s00449-014-1165-x)
Supplement: Supplementary file 1 — Supplementary material 1 (DOCX 19 kb) [file 449_2014_1165_MOESM1_ESM.docx]

**List of supplementary tables**

**S 1. Compositional changes of rice straw in 30 independent pretreatment conditions**

| Test No. | Cellulose  (wt%) | Hemicellulose  (wt%) | Lignin  (wt%) | Ash  (wt%) |
| --- | --- | --- | --- | --- |
| Not pretreated | 31.8 | 17.5 | 18.2 | 6.9 |
| 1 | 46.2 | 24.4 | 13.9 | 12.7 |
| 2 | 50.8 | 19.8 | 14.1 | 11.8 |
| 3 | 48.5 | 21.8 | 14.1 | 12.3 |
| 4 | 52.1 | 17.8 | 14.3 | 13.8 |
| 5 | 48.8 | 23.3 | 13.1 | 12.5 |
| 6 | 53.0 | 18.0 | 11.7 | 12.9 |
| 7 | 50.5 | 20.7 | 12.2 | 12.6 |
| 8 | 53.2 | 18.3 | 12.9 | 14.5 |
| 9 | 49.6 | 26.9 | 14.2 | 11.1 |
| 10 | 57.6 | 22.0 | 12.7 | 12.0 |
| 11 | 45.4 | 23.7 | 15.2 | 13.0 |
| 12 | 49.9 | 18.6 | 15.5 | 14.9 |
| 13 | 51.7 | 24.8 | 13.1 | 10.4 |
| 14 | 53.5 | 17.2 | 12.5 | 14.0 |
| 15 | 47.6 | 22.9 | 14.6 | 12.5 |
| 16 | 52.9 | 17.8 | 13.8 | 14.1 |
| 17 | 46.8 | 23.1 | 14.4 | 11.7 |
| 18 | 55.2 | 15.8 | 12.4 | 13.8 |
| 19 | 48.7 | 23.5 | 12.0 | 12.6 |
| 20 | 51.5 | 19.5 | 13.4 | 13.3 |
| 21 | 41.5 | 22.3 | 19.9 | 10.7 |
| 22 | 49.2 | 19.0 | 12.3 | 14.3 |
| 23 | 55.8 | 21.6 | 7.3 | 12.5 |
| 24 | 47.1 | 19.7 | 13.8 | 12.8 |
| 25 | 48.0 | 19.6 | 13.2 | 13.6 |
| 26 | 50.3 | 20.0 | 14.0 | 13.0 |
| 27 | 47.9 | 19.9 | 13.2 | 12.4 |
| 28 | 50.1 | 19.7 | 13.5 | 12.8 |
| 29 | 47.9 | 19.8 | 12.6 | 12.8 |
| 30 | 50.9 | 19.6 | 13.5 | 12.5 |

**S 2. Glucose conversion rate by enzymatic hydrolysis in the 30 independent pretreatment conditions**

| Test No. | Moisture content  (%) | Glucan  concentration in hydrolysate  (%) | Hydrolysate containing 0.1 g of glucan  (g) | Weight of glucan measured  (g) | Added 0.1 M citrate buffer  at pH 5.0  (ml) | Added water for 10 ml working volume (ml) | Glucan concentration  (g/l) | Total glucose conversion  (%) |
| --- | --- | --- | --- | --- | --- | --- | --- | --- |
| Avicel | 4.80 | 0.1106 | 0.1108 | 0.1002 | 5.0 | 4.62 | 10.02 | 97.32 |
| 1 | 7.69 | 0.2344 | 0.2347 | 0.1001 | 5.0 | 4.50 | 10.01 | 77.99 |
| 2 | 7.01 | 0.2116 | 0.2118 | 0.1001 | 5.0 | 4.52 | 10.01 | 90.19 |
| 3 | 7.33 | 0.2223 | 0.2226 | 0.1001 | 5.0 | 4.51 | 10.01 | 84.46 |
| 4 | 6.95 | 0.2063 | 0.2062 | 0.1000 | 5.0 | 4.53 | 10.00 | 89.16 |
| 5 | 7.84 | 0.2223 | 0.2223 | 0.1000 | 5.0 | 4.51 | 10.00 | 90.48 |
| 6 | 7.60 | 0.2041 | 0.2041 | 0.1000 | 5.0 | 4.53 | 10.00 | 99.04 |
| 7 | 7.57 | 0.2143 | 0.2147 | 0.1002 | 5.0 | 4.52 | 10.02 | 93.39 |
| 8 | 7.33 | 0.2030 | 0.2039 | 0.1005 | 5.0 | 4.53 | 10.05 | 93.12 |
| 9 | 7.73 | 0.2186 | 0.2182 | 0.0998 | 5.0 | 4.51 | 9.98 | 69.36 |
| 10 | 7.36 | 0.1874 | 0.1879 | 0.1003 | 5.0 | 4.55 | 10.03 | 69.05 |
| 11 | 8.03 | 0.2393 | 0.2398 | 0.1002 | 5.0 | 4.49 | 10.02 | 81.96 |
| 12 | 7.54 | 0.2166 | 0.2163 | 0.0999 | 5.0 | 4.52 | 9.99 | 92.81 |
| 13 | 7.67 | 0.2095 | 0.2098 | 0.1001 | 5.0 | 4.52 | 10.01 | 79.87 |
| 14 | 6.87 | 0.2007 | 0.2001 | 0.0997 | 5.0 | 4.53 | 9.97 | 97.18 |
| 15 | 7.44 | 0.2272 | 0.2275 | 0.1002 | 5.0 | 4.51 | 10.02 | 88.04 |
| 16 | 7.66 | 0.2046 | 0.2047 | 0.1000 | 5.0 | 4.53 | 10.00 | 98.62 |
| 17 | 7.51 | 0.2313 | 0.2312 | 0.1000 | 5.0 | 4.50 | 10.00 | 84.80 |
| 18 | 5.10 | 0.1908 | 0.1907 | 0.1000 | 5.0 | 4.54 | 10.00 | 97.48 |
| 19 | 7.80 | 0.2225 | 0.2227 | 0.1001 | 5.0 | 4.51 | 10.01 | 87.43 |
| 20 | 7.73 | 0.2103 | 0.2106 | 0.1001 | 5.0 | 4.52 | 10.01 | 93.04 |
| 21 | 7.60 | 0.2610 | 0.2617 | 0.1003 | 5.0 | 4.47 | 10.03 | 46.75 |
| 22 | 6.83 | 0.2182 | 0.2181 | 0.0999 | 5.0 | 4.52 | 9.99 | 97.82 |
| 23 | 7.12 | 0.1930 | 0.1933 | 0.1001 | 5.0 | 4.54 | 10.01 | 96.00 |
| 24 | 7.48 | 0.2294 | 0.2297 | 0.1002 | 5.0 | 4.50 | 10.02 | 95.95 |
| 25 | 7.76 | 0.2257 | 0.2257 | 0.1000 | 5.0 | 4.51 | 10.00 | 97.12 |
| 26 | 6.82 | 0.2135 | 0.2132 | 0.0999 | 5.0 | 4.52 | 9.99 | 88.76 |
| 27 | 7.69 | 0.2262 | 0.2269 | 0.1003 | 5.0 | 4.51 | 10.03 | 96.37 |
| 28 | 7.50 | 0.2157 | 0.2155 | 0.0999 | 5.0 | 4.52 | 9.99 | 85.44 |
| 29 | 7.77 | 0.2261 | 0.2260 | 0.0999 | 5.0 | 4.51 | 9.99 | 97.60 |
| 30 | 7.63 | 0.2128 | 0.2126 | 0.0999 | 5.0 | 4.52 | 9.99 | 87.64 |
